# Unlocking Nature’s Defense: Plant Pattern Recognition Receptors as Guardians Against Pathogenic Threats

**DOI:** 10.1094/MPMI-10-23-0177-HH

**Published:** 2024-02-28

**Authors:** Chao Zhang, Yingpeng Xie, Ping He, Libo Shan

**Affiliations:** Department of Molecular, Cellular, and Developmental Biology, University of Michigan, Ann Arbor, MI 48109, U.S.A.

**Keywords:** danger-triggered immunity (DTI), effector-triggered immunity (ETI), pathogen infection, pattern-triggered immunity (PTI), plant immunity, receptor kinases (RKs), receptor proteins (RPs)

## Abstract

Embedded in the plasma membrane of plant cells, receptor kinases (RKs) and receptor proteins (RPs) act as key sentinels, responsible for detecting potential pathogenic invaders. These proteins were originally characterized more than three decades ago as disease resistance (R) proteins, a concept that was formulated based on Harold Flor’s gene-for-gene theory. This theory implies genetic interaction between specific plant R proteins and corresponding pathogenic effectors, eliciting effector-triggered immunity (ETI). Over the years, extensive research has unraveled their intricate roles in pathogen sensing and immune response modulation. RKs and RPs recognize molecular patterns from microbes as well as dangers from plant cells in initiating pattern-triggered immunity (PTI) and danger-triggered immunity (DTI), which have intricate connections with ETI. Moreover, these proteins are involved in maintaining immune homeostasis and preventing autoimmunity. This review showcases seminal studies in discovering RKs and RPs as R proteins and discusses the recent advances in understanding their functions in sensing pathogen signals and the plant cell integrity and in preventing autoimmunity, ultimately contributing to a robust and balanced plant defense response.

Being immobile, plants are constantly exposed to a myriad of pathogenic threats. Without adaptive immunity and specialized immune cells, plants have developed an extensive repertoire of receptor kinases (RKs) and proteins (RPs) that act as “border controls,” monitoring the presence of pathogenic invaders (Bender and Zipfel 2023; Ge et al. 2022; Zhang et al. 2023).

Members of RKs and RPs function as pattern recognition receptors (PRRs), which sense microbe-associated molecular patterns (MAMPs) in mediating pattern-triggered immunity (PTI). RKs and RPs possess a transmembrane domain mediating the plasma membrane localization. In addition, RKs and RPs bear diverse extracellular domains, including leucine-rich repeats (LRRs), lysine motifs (LysM), lectin motifs, malectin-like motifs, and epidermal growth factor (EGF)-like repeats, which mainly mediate the MAMP recognition. Unlike RKs, RPs lack the intracellular kinase domain and therefore, often form a complex with RKs in relaying their intracellular signaling (Escocard de Azevedo Manhães et al. 2021).

Meanwhile, some pathogens can overcome plant PTI by secreting effector proteins, which results in effector-triggered susceptibility. In turn, plants make use of intracellular nucleotide-binding site leucine-rich repeat (NBS-LRR) receptors (NLRs) to recognize effectors directly or indirectly, cumulating in effector-triggered immunity (ETI) (Ngou et al. 2022). The ETI response typically involves rapid programmed cell death occurring at the initial pathogen infection site, a phenomenon referred to as the hypersensitive response (HR) (Remick et al. 2023).

Pathogen infection also changes plant cell integrity and induces the production of modified self-components as danger-associated molecular patterns (DAMPs) and phytocytokines in eliciting danger-triggered immunity (DTI) (Gust et al. 2017; Tanaka and Heil 2021; Zhang et al. 2023). PTI and DTI often trigger a series of shared signaling events and deploy some common immune components and signaling pathways (Hou et al. 2021; Tanaka and Heil 2021). In addition, DTI either amplifies or modulates PTI (Ortiz-Morea et al. 2022). Furthermore, the boundary between PTI and ETI classifications is becoming increasingly blurred, as the pathways depend on each other, share signaling components, and reciprocally potentiate for a robust defense against pathogens (Ngou et al. 2021; Pruitt et al. 2021; Tian et al. 2021; Yuan et al. 2021a). In addition, RK- and RP-mediated signaling pathways play a role in regulating the strength and duration of the immune response in maintaining immune homeostasis (Ortiz-Morea et al. 2022).

In this review, we will highlight some momentous discoveries regarding the roles of RKs and RPs initially characterized as R proteins in mediating ETI and later recognized as PRRs in eliciting PTI and DTI. We will also delve into recent advances in understanding the coordination of PTI and ETI through DTI mediated by RKs or RPs to establish a robust immune system in fending off pathogen infections. Furthermore, we will discuss the contributions of RKs and RPs in attenuating immune responses and explore emerging insights into the concerted actions of PTI, DTI, and ETI to achieve immune homeostasis.

## RKs and RPs Were Initially Identified as Disease Resistance (R) Proteins

**Fig. 1.**
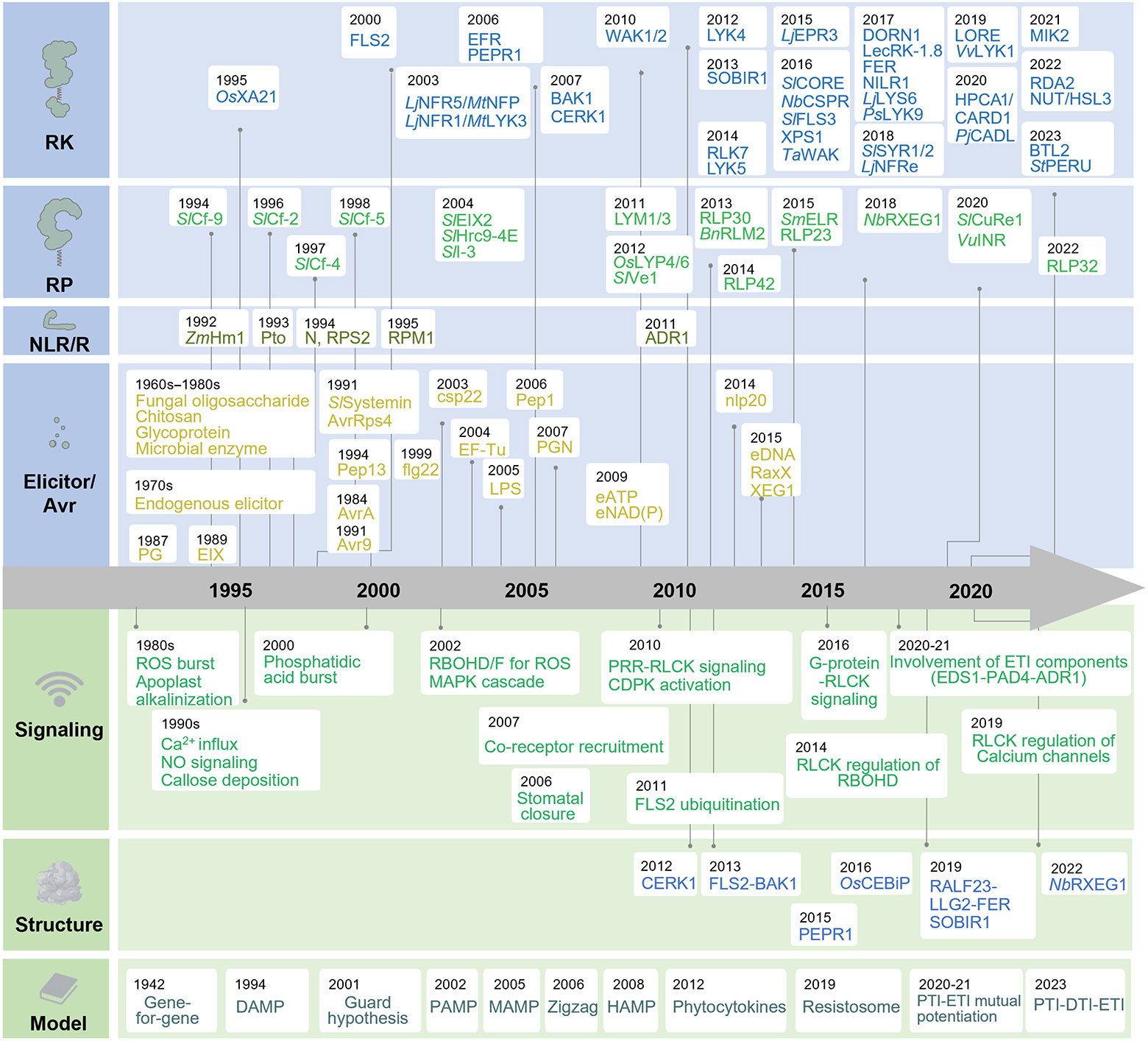
Timeline of the studies of receptor kinases/receptor proteins (RKs/RPs) in plant immunity. Some representative studies of RKs and RPs in plant immunity are chronically highlighted along with identifications of some earliest nucleotide-binding site leucine-rich repeat receptors (NLRs) and R proteins, pathogen elicitors, pathogen-associated molecular patterns (PAMPs)/microbe-associated molecular patterns (MAMPs)/danger-associated molecular patterns (DAMPs)/phytocytokines, and avirulence factors to showcase Flor’s gene-for-gene theory in molecular plantpathogen interaction. Important signaling events and components in pattern-triggered immunity (PTI)/danger-triggered immunity (DTI), structural analyses of RKs/RPs, and models of plant immunity are included to highlight complex mechanisms underlying pathogen recognition and plantpathogen coevolution. Please note that because of space limitations, not all relevant works are mentioned. ADR1, ACTIVATED DISEASE RESISTANCE 1; Avr, avirulence; BAK1, BRASSINOSTEROID-INSENSITIVE 1-ASSOCIATED KINASE 1; BTL2, BAK-TO-LIFE 2; CADL, CANNOT RESPOND TO DMBQ-LIKE PROTEIN; CARD1, CANNOT RESPOND TO DMBQ (2,6-dimethoxy-1,4-benzoquinone) 1; CDPK, calcium-dependent protein kinases; CEBiP, CHITIN ELICITOR-BINDING PROTEIN; CERK1, CHITIN ELICITOR RECEPTOR KINASE 1; Cf-9, *Cladosporium fulvum* resistance-9; CORE, cold shock protein receptor; CSPR, RECEPTOR-LIKE PROTEIN REQUIRED FOR CSP22 RESPONSIVENESS; CuRe1, CUSCUTA RECEPTOR 1; DORN1, DOES NOT RESPOND TO NUCLEOTIDES; EDS1, ENHANCED DISEASE SUSCEPTIBILITY 1; EFR, EF-Tu RECEPTOR; EIX, ethylene-inducing xylanase; ELR, elicitin response; EPR3, EXOPOLYSACCHARIDE RECEPTOR 3; ETI, effector-triggered immunity; FER, FERONIA; FLS2, FLAGELLIN SENSING 2; FLS3, FLAGELLIN-SENSING 3; HAMP, herbivore-associated molecular pattern; Hcr9-4E, homologues of *Cladosporium* resistance gene Cf-9 4E; HM1, *Helminthosporium carbonum* susceptibility 1; HPCA1, HYDROGEN-PEROXIDE-INDUCED Ca^2+^ INCREASES 1; HSL3, HAESA-LIKE 3; INR, inceptin receptor; LecRK, LECTIN RECEPTOR KINASE; LLG2, LORELEI-LIKE-GPI-ANCHORED PROTEIN 2; LORE, LIPOOLIGOSACCHARIDE-SPECIFIC REDUCED ELICITATION; LPS, lipopolysaccharides; LYK, LysM-CONTAINING RECEPTOR-LIKE KINASE; LYK3, LYSIN-MOTIF-RECEPTOR LIKE KINASE 3; LYP, LysM-containing protein; MAPK, mitogen-activated protein kinase; MIK2, LRR-RK MALE DISCOVERER 1-INTERACTING RECEPTOR LIKE KINASE 2; NFP, NOD-FACTOR PERCEPTION; NFR1/NFR5, NOD FACTOR RECEPTORS 1 and 5; NILR1, NEMATODE-INDUCED LRR-RLK 1; NO, nitric oxide; PAD4, PHYTOALEXIN DEFICIENT 4; PEPR1, PEP RECEPTOR 1; PERU, Pep-13 receptor unit; PG, peptidoglycan; PGN, peptidoglycan; RALF, RAPID ALKALINIZATION FACTOR; RaxX, XA21-mediated immunity X; RBOHD, respiratory burst oxidase homolog D; RDA2, RESISTANT TO DFPM-INHIBITION OF ABSCISIC ACID SIGNALING 2; RLCK, receptor-like cytoplasmic kinase; ROS, reactive oxygen species; RPM1, resistance to *Pseudomonas syringae* pv*. maculicola* 1; RPS2, RESISTANT TO *P. syringae* 2; RXEG1, Response to XEG1; SOBIR1, SUPPRESSOR OF BAK1-INTERACTING RECEPTOR-LIKE KINASE 1-1; SYR1/2, SYSTEMIN RECEPTOR 1 and 2; WAK, WALL-ASSOCIATED KINASE; XA21, *Xanthomonas oryzae* pv. *oryzae* RESISTANCE 21; XPS1, XANTHINE/PERMEASE SENSING 1.

Harold Flor’s research in the early twentieth century on the genetic interplay between flax and the flax rust fungus demonstrated that disease resistance is conferred through specific interactions between plant and pathogen genes (Flor 1942, 1955). In the 1990s, with the advance of molecular genetics, a series of plant disease resistance genes were identified, including genes encoding a detoxifying enzyme HM1 (*Helminthosporium carbonum* SUSCEPTIBILITY 1) (Johal and Briggs 1992), intracellular kinase Pto (*Pseudomonas syringae* pv. *tomato*) (Martin et al. 1993), intracellular NBS-LRR proteins RPS2 (RESISTANT TO *P. syringae* 2) (Bent et al. 1994; Mindrinos et al. 1994), RPM1 (RESISTANCE TO *Pseudomonas syringae* pv*. maculicola* 1) (Grant et al. 1995) and N proteins (*Tobacco mosaic virus* RESISTANCE PROTEIN N) (Whitham et al. 1994), plasma membrane-resident RP Cf-9 (*Cladosporium fulvum* RESISTANCE-9) (Jones et al. 1994), and RK XA21 (*Xanthomonas oryzae* pv. *oryzae* RESISTANCE 21) (Song et al. 1995) (Fig. 1). Among those, NBS-LRR proteins are often considered conventional R proteins, which recognize pathogen race-specific effectors either directly or indirectly, whereas other kinases and enzymes can be viewed as exceptional cases (Duxbury et al. 2021).

The rice (*Oryza sativa*) *OsXA21* gene, which confers resistance to *X. oryzae* pv. *oryzae*, a bacterial pathogen that causes rice bacterial blight disease, was isolated using classical positional cloning techniques (Song et al. 1995). This landmark discovery marked the identification of one of the first *RK* genes involved in plant disease resistance. *Os*XA21 falls within the clade of RKs carrying an extracellular LRR domain, a single transmembrane, and a cytosolic kinase domain, collectively referred to as LRR-RKs. This clade encompasses a significant number of family members, with many playing crucial roles in plant defense responses (Albert et al. 2020; Bender and Zipfel 2023; Escocard de Azevedo Manhães et al. 2021). *Os*XA21 recognizes and binds to sulfated peptide RaxX (REQUIRED FOR ACTIVATION OF *XA21*-MEDIATED IMMUNITY X) from *X. oryzae* pv. *oryzae* (Luu et al. 2019; Pruitt et al. 2015). Intriguingly, a wide collection of *X. oryzae* pv. *oryzae* field strains encode the *raxSTAB* (*raxST*, *raxA*, and *raxB*) operon, which is required for triggering *Os*XA21-mediated resistance (Pruitt et al. 2015). This contrasts with the pathogen race-specific resistance mediated by classical *R* genes. Interestingly, RaxX also promotes plant growth, mimicking the function of some plant peptide hormones and likely benefiting the early stage of biotrophic growth of pathogens (Pruitt et al. 2017). The identification of rice *Os*XA21 and *X. oryzae* pv. *oryzae* RaxX, and the elucidation of their molecular and biochemical binding shed light on the genetic and biochemical basis of Flor’s gene-for-gene theory and underscore the importance of specific molecular recognition in plant–pathogen interactions.

The tomato (*Solanum lycopersicum*) *Sl*Cf-9 is the first RP identified and cloned for resistance to leaf mold fungal pathogen *Cladosporium fulvum* (*Cf*) by transposon tagging (Jones et al. 1994). *Sl*Cf-9 recognizes the pathogen-secreted apoplastic peptide effector Avr9 (Avirulence 9), which was named based on its ability to trigger an avirulence response in resistant tomato lines (Jones et al. 1994; Van den Ackerveken et al. 1992). Since then, a series of other *Sl*Cf proteins from tomato, including *Sl*Cf-2, *Sl*Cf-4, *Sl*Cf-5, and *Sl*Cf-19, have been identified to recognize cognate pathogen race-specific effectors, including Avr2, Avr4, and Avr5 (Dixon et al. 1996, 1998; Takken et al. 1999; Thomas et al. 1997; Zhao et al. 2016) (Fig. 1). The molecular interaction between *Sl*Cfs and Avr proteins is a showcase of the specificity of the gene-for-gene relationship. Notably, pathogen avirulent proteins also possess virulent functions in promoting pathogenicity, and such dual functions of a single avirulent protein can be uncoupled via different motifs, which may be recognized by distinct plant receptors mediating either resistance or susceptibility (Shan et al. 2000, 2008).

All *Sl*Cfs carry an extracellular LRR domain, similar to the LRR-RK *Os*XA21, but they lack the intracellular kinase domain. The variant parts confined to LRR regions in *Sl*Cfs determine the genetic recognition specificities toward the corresponding avirulent effectors (Van der Hoorn et al. 2001). It is worth noting that *Sl*Cfs do not interact directly with cognate Avrs but rather associate with them, likely through other components within the complex, to trigger immune signaling (Liebrand et al. 2014; Luderer et al. 2001). In addition, both *Os*XA21 and *Sl*Cfs, while unconventional in that they do not belong to the NBS-LRR protein class, share similarities with NBS-LRR proteins in their ability to induce HR and trigger pathogen race-specific disease resistance (Jones et al. 1994; Pruitt et al. 2015), in contrast to other PRRs not normally triggering HR. This further infers the blurred boundary between PTI and ETI.

## RKs and RPs Function as PRRs Sensing MAMPs

Flor’s theory, which emphasizes the specific interaction between pathogen *Avr* gene and host *R* gene, seems contradictory to the identification of general microbial elicitors that trigger immune responses in various plant species. For instance, proteins from diverse bacterial flagella, like flagellin or its synthetic flg22 peptides, as well as fungal cell wall components, like chitin oligosaccharides, can trigger immune responses in different plant species (Felix et al. 1999; Shibuya and Minami 2001). Subsequent identifications of the receptors responsible for perceiving these general elicitors from pathogens led to the introduction of the terms “PAMP” (pathogen-associated molecular pattern) and “PRR” in the field of plant–microbe interactions, in parallel with studies in vertebrate immunity (Nürnberger and Brunner 2002). To account for the presence of conserved patterns in both pathogenic and nonpathogenic microbes, the term “MAMP” was also proposed for these elicitors (Ausubel 2005).

With the conceptual advancement of MAMPs and PRRs and the implementation of diverse genetic and biochemical tools in the study of plant–microbe interactions, significant progress has been achieved in identifying various MAMPs and isolating their cognate PRRs (Fig. 1). These studies have not only profoundly expanded our knowledge of a wide array of MAMP–PRR pairs but have also prompted significant inquiries regarding their recognition specificities and conservation (Kong et al. 2021; Zhang et al. 2023).

In this section, we highlight pioneering efforts in the traditional genetic and biochemical isolation of RKs and RPs as PRRs. We then discuss genomics-assisted methods for identifying PRRs and uncovering the complexity of their recognition specificities.

### Classical genetic and biochemical isolations of RKs and RPs as PRRs

FLS2 (FLAGELLIN SENSING 2) is an LRR-RK whose encoding gene was discovered using the positional cloning technique, like that used to identify *OsXA21* in rice. This discovery involved studying *Arabidopsis* ecotypes bearing different responses to flg22, establishing FLS2 as the most extensively studied PRR perceiving bacterial flagellin in plants (Gómez-Gómez and Boller 2000). The LRR-RK EFR (EF-Tu RECEPTOR) that recognizes bacterial EF-Tu (ELONGATION FACTOR-Tu) or the cognate synthetic peptide elf18, was identified by a targeted forward genetic approach toward mutants of transcriptionally induced *RKs* upon elf18 treatment (Kunze et al. 2004; Zipfel et al. 2006). Remarkably, following the perception of MAMP, numerous LRR-RKs, including notable examples like FLS2 and EFR, recruit BAK1 (BRASSINOSTEROID-INSENSITIVE 1-ASSOCIATED KINASE 1) as a co-receptor to relay signaling (Chinchilla et al. 2007; Ma et al. 2016). BAK1, also recognized as SERK3 (SOMATIC EMBRYOGENESIS RECEPTOR-LIKE KINASE 3), is a family member of five LRR-RKs in *Arabidopsis* (Ma et al. 2016). SERKs directly complex with diverse LRR-RKs in perceiving MAMPs and plant endogenous peptides involved in triggering plant DTI and regulating plant growth and development (Ma et al. 2016).

An LysM-containing RP *Os*CEBiP (CHITIN ELICITOR-BINDING PROTEIN) from rice was isolated via biochemical affinity purifications as the fungal chitin receptor based on its strong binding to chitin oligomers (Miya et al. 2007). Notably, unlike other classical RPs, *Os*CEBiP does not have a transmembrane structure; instead, it localizes to the plasma membrane via a glycosylphosphatidylinositol (GPI) anchor (Gong et al. 2017). Following the identification of *Os*CEBiP, other LysM-containing RKs, also known as LYKs, including CERK1 (CHITIN ELICITOR RECEPTOR KINASE 1), were discovered to be involved in chitin perception and signaling in rice and *Arabidopsis* (Shimizu et al. 2010; Wan et al. 2012). In *Arabidopsis*, LYK4 and LYK5 bind and perceive chitin oligomers in triggering the immune signaling (Cao et al. 2014; Petutschnig et al. 2010; Wan et al. 2012). Additionally, CERK1 plays a role in immune responses triggered by various MAMPs, including β-1,3/1,4-glucans, the primary cell wall glucans in fungi (Rebaque et al. 2021; Yang et al. 2021), bacterial lipopolysaccharide (Desaki et al. 2018), and peptidoglycans (Willmann et al. 2011), and plant DAMP oligogalacturonides (Wang et al. 2020). Unlike LYK4 and LYK5, CERK1 bears a strong kinase activity (Cao et al. 2014; Wan et al. 2012), likely for initiating the intracellular signaling upon chitin perception by LYK4 and LYK5. Much like the BAK1/SERKs, which act as co-receptors for multiple LRR-RKs and LRR-RPs, CERK1 likely functions as a co-receptor, forming complexes with distinct LysM receptors in response to the perception of specific polysaccharides.

### Recognition specificity and evolution of RKs/RPs as PRRs in recognizing MAMPs

The capacity of bulk sequencing and genome assembling, along with the availability of diverse plant varieties/ecotypes/species and genetic tools, has greatly facilitated the identification of long-sought PRRs. The examination of PRRs in different plant species and their cognate MAMPs in different pathogens also provides insight into the evolutionary perspectives of MAMP–PRR recognition specificity. Pep (Plant elicitor peptide) 13, a 13 conserved amino acid peptide, was originally identified within an abundant cell wall glycoprotein (GP42) from *Phytophthora sojae* for its ability to trigger an unknown receptor-mediated defense response in parsley (Nürnberger et al. 1994). GP42 was further shown to be a *P. sojae* cell wall-associated Ca^2+^-dependent TGase (*R*-glutaminyl-peptide:amine-γ-glutamyltransferase) (Brunner et al. 2002). Interestingly, the same amino acid residues in Pep13 required for triggering the immune response in parsley and potato are also required for TGase activity (Brunner et al. 2002). Pep13 is widespread and highly conserved among plant-associated oomycetes (Brunner et al. 2002). This implies that plants may have evolved receptors that recognize conserved molecular motifs or patterns present within and essential for the function of pathogen-derived molecules. A recent work using bulk-segregant RNA sequencing along with genetic mapping of the backcrossed populations has identified the potato (*Solanum tuberosum*) LRR-RK *St*PERU (Pep13 RECEPTOR UNIT) as the receptor sensing Pep13 (Torres Ascurra et al. 2023). *St*PERU binds Pep13 and enhances plant immunity to *P. infestan* infections (Torres Ascurra et al. 2023), supporting PERU as the bona fide receptor for Pep13. Alanine scanning mutagenesis of Pep13 for its defense inducibility on a wide collection of potato species reveals the vast functional diversification of PERU. Geographic distribution and genetic variation analysis reveal the evolution of PERU and Pep13 ligand binding diversification among potato species (Lin et al. 2023; Torres Ascurra et al. 2023). The functional divergence of PERU is likely driven by MAMP Pep13 mutations. It will be interesting to survey the genetic diversity of the Pep13 patterns in diverse oomycete strains from the field and examine how the pathogen Pep13 pattern changes may shape the plant PEPR function diversification.

Besides the small peptide epitope, proteobacterial translation initiation factor 1 (IF1) is recognized by the *Arabidopsis* LRR-RP RLP32. Notably, RLP32 activation requires the full-length IF1 protein (Fan et al. 2022). This implies that unlike the direct binding of PRR with cognate ligands, likely FLS2 to flg22 and PERU to Pep13, tertiary fold features of ligands may be required for PRR recognition.

The recognition of MAMPs by PRRs typically exhibits specificity within limited host–pathogen interactions. This specificity can extend to cross-kingdom MAMP–PRR recognitions from either pathogens or hosts. While XEG1-type glucanohydrolases are found in various plant pathogenic fungi and oomycetes, their cognate receptors have only been identified in solanaceous plants (Ma et al. 2015). Similarly, the homologs of *Vm*E02, a small cysteine-rich protein from the necrotrophic fungus *Valsa mali*, are present in diverse oomycetes and fungi in triggering plant immune response (Nie et al. 2019). Some convergently evolved PRRs may sense MAMPs from different microbial kingdoms. Interestingly, SCP (SMALL CYSTEINE-RICH PROTEIN), also present in both fungi and oomycetes, can be recognized as a ligand by RLP30 in *Arabidopsis*, by RLP *Nb*RE02 (RESPONSE TO *Vm*E02) in *Nicotiana benthamiana*, and by an unidentified *Brassica* PRR (Yang et al. 2023). This implies further expanded recognition capabilities among diverse plant and microbe species. Intriguingly, these receptors exhibit limited sequence similarity, and RLP30 also can recognize another SCP-nonhomologous protein secreted by the bacterial pathogen *Pseudomonas* (Yang et al. 2023). Moreover, with limited sequence similarity, RLP30 and *Nb*RE02 respond to different parts of SCP proteins (Yang et al. 2023). This indicates that different PRRs may employ distinct recognition mechanisms in recognizing the same MAMP ligand.

## RKs and RPs Sense Phytocytokines for DTI

As an integral part of the plant immune system, RKs and RPs have also evolved to recognize disrupted self-components during infections. These disruptions occur when the plant cell integrity is compromised, leading to the production of DAMPs (Gust et al. 2017; Tanaka and Heil 2021; Yu et al. 2017). Phytocytokines are small immunomodulatory peptides that are perceived by RKs and RPs to either enhance or attenuate plant immunity (Hou et al. 2021; Tanaka and Heil 2021).

### Phytocytokines produced during infections and plant growth and development

The production of phytocytokines is regulated at both transcriptional and translational levels in response to infections. Upon attacks by pathogens, herbivores, mechanical damage, or MAMP treatment, the genes encoding precursor proteins of phytocytokines and their cognate receptors are often transcriptionally upregulated at either infection sites or adjacent tissues, likely as a result of a positive feedback circuit for amplification or modulation of the plant defense responses. Some phytocytokine-encoding genes are transcriptionally upregulated by environmental abiotic stresses in addition to pathogen infections. For instance, the expressions of *SCREW*s (*SMALL PHYTOCYTOKINES REGULATING DEFENSE AND WATER LOSS*s) and their corresponding LRR-RK receptor *NUT* gene are induced by various stresses, including drought, sap-sucking insect aphids, MAMP treatment, and pathogen infections (Liu et al. 2022; Rhodes et al. 2022b). In line with this, SCREWs-NUT plays a pivotal role in regulating stomal movement and apoplast water potential through plant hormone abscisic acid-mediated signaling, enhancing plant drought tolerance and resistance to both bacterial infections and insect infestations (Guzel Deger et al. 2015; Liu et al. 2022). It remains elusive in terms of the mechanisms underlying their spatiotemporal transcriptional regulation in response to different stress factors.

Phytocytokines usually contain a signal peptide at the amino (N)-terminus of the precursor proteins for secretion. However, some phytocytokines without a signal peptide can be released from the damaged cells into the apoplast (Hou et al. 2021; Matsubayashi 2014). Phytocytokines are derived from large precursor proteins via a maturation process involving proteolytic cleavage or posttranslational modifications, and are subsequently released into the apoplast, the extracellular space, where they are recognized by the ectodomains of RKs and RPs. The maturation of certain phytocytokines, like Pep1 induced by damage, is mediated by the calcium-dependent proteases known as metacaspases (MCs) (Hander et al. 2019; Shen et al. 2019). Without infections, PROPEP1, the precursor protein of Pep1 is associated with the tonoplast, whereas MC4 remains dormant in the cytosol. MAMP sensing or the disruption of the plasma membrane integrity leads to a calcium influx, thereby activating MC4, which cleaves PROPEP1 (Zhu et al. 2020). Subsequently, Pep1 is released from the cytosol into the apoplast, where it binds to the cognate receptor complex consisting of the LRR-RKs PEPR1 (PEP RECEPTOR 1) and PEPR2 in complex with BAK1 (Hander et al. 2019; Shen et al. 2019). It is not yet entirely clear whether phytocytokines like Pep1 are actively secreted or passively released into the apoplast because of disruptions in the plasma membrane integrity. Whether Peps can trigger longdistance defense signaling also remains a mystery. The precise mechanisms governing the transcriptional regulation, production, maturation, and secretion of most phytocytokines in response to diverse biotic and abiotic stresses are still a subject of ongoing research investigation.

Some phytocytokines were originally found to play an important role in regulating different aspects of plant growth and development. For instance, the peptide IDA (INFLORESCENCE DEFICIENT IN ABSCISSION), perceived by the receptor LRR-RK HAE (HAESA) and HSL2 (HAESA-LIKE2) and co-receptor BAK1/SERKs, regulates floral organ abscission and lateral root emergence (Wang et al. 2021; Zhu et al. 2019). IDA also triggers a series of typical PTI signaling events and expression of defense-related genes (Lalun et al. 2023; Patharkar et al. 2017). The IDA-HAE/HSL/BAK1/SERKs-mediated immune signaling may protect against pathogen infection during cell separation. RGFs (ROOT MERISTEM GROWTH FACTORS), also known as GLVs (GOLVENs), involved in root meristem development and maintenance, are recognized by LRR-RK RGIs (ROOT MERISTEM GROWTH FACTOR INSENSITIVEs) (Matsuzaki et al. 2010). The expression of *RGF7* precursor gene *PRORGF7* is strongly upregulated upon flg22 treatment. Inducible overexpression of *PRORGF7* triggers RGI4- and RGI5-dependent PTI responses (Wang et al. 2021). In another case, RGF9, recognized by RGI3, enhances bacterial resistance by increasing FLS2 and EFR protein levels independent of transcriptional regulation (Stegmann et al. 2022). It remains unknown how RGF9/RGI3 contributes mechanistically to the increased protein levels of different PRRs. Notably, these phytocytokine precursor-encoding genes often possess unique tissue expression patterns related to specific stages of plant growth and development or upon infection, while *BAK1/SERKs* are abundantly expressed in various tissues across different developmental stages. Thus, transcriptional regulation of phytocytokine precursor-encoding genes may partially explain their specific involvement in plant immunity, growth, and development.

### Phytocytokines potentiate MAMP-triggered signaling and immunity

Phytocytokines trigger largely overlapping signaling events as MAMPs, including recruitment of co-receptor BAK1/SERK4, phosphorylation of receptor-like cytosolic kinase BIK1 (BOTRYTIS-INDUCED KINASE 1), activation of mitogen-activated protein kinase (MAPK), an increase in cytosolic calcium concentration, reactive oxygen species (ROS) burst, and stomatal closure. Pretreatment of phytocytokines can enhance plant resistance against subsequent infections. Additionally, RKs or RPs sensing phytocytokines are required for the full activation of MAMP-triggered signaling and immunity. Thus, phytocytokine-mediated signaling is often viewed as an intrinsic mechanism to amplify MAMP-triggered signaling.

Certain phytocytokines and their cognate RK and RP receptors are in the same complex with MAMP receptors and BAK1/SERK4 co-receptors and can modulate the MAMP receptor–co-receptor complex stability. FER (FERONIA) in coordination with a GPI-anchored protein LLG1 (LORELEI-LIKE GPI-ANCHORED PROTEIN 1) facilitates the MAMP-induced PRR complex formation. While FER weakly interacts with FLS2 and BAK1, its association with BAK1 is enhanced upon flg22 treatment. Notably, FER is necessary for the flg22-induced FLS2-BAK1 complex formation (Stegmann et al. 2017). Additionally, FER-mediated PRR complex formation and PTI responses are independent of its kinase activity (Gronnier et al. 2022). These findings raise further questions about the mechanism underlying FER-mediated MAMP receptor complex formation and immune-promoting function.

Notably, FER appears to exert diverse functions by sensing different ligands, in addition to multiple members of RALFs for opposing signaling outputs in plant immunity, in coordinating plant growth and various abiotic stresses. For instance, FER binds pectin, a major component of primary cell walls, to directly activate ROP6 (RHO OF PLANTS 6) guanosine triphosphatase (GTPase) to regulate pavement cell morphogenesis (Haruta et al. 2014; Lin et al. 2022). FER senses cell wall disruptions, leading to the production of cell wallmodifying enzymes and oligosaccharides, which in turn trigger defense responses and negatively affect plant growth through EGF domain-containing RK WAKs (WALL-ASSOCIATED KINASES), which also bind to cell wall pectin (Liu et al. 2023). The direct sensing of cell wall pectin by FER also plays a key role in the plant response to salt stress (Feng et al. 2018). Upon detecting the mechanical and/or biochemical effects of high salt on cell walls, FER initiates a transient and cell type-specific calcium peak triggering the mechanical reinforcement of the cell wall (Feng et al. 2018). These observations support the versatile role of FER in connecting the plant cell wall integrity by binding to pectin with the plasma membrane-resident PRR complexes.

As mentioned earlier, RGI4 and RGI5 perceive RGF7 and form flg22-induced complex with co-receptor BAK1, which is also required for RGF7-induced PTI responses (Wang et al. 2021). In addition, RGI3 associates and complexes with FLS2 upon flg22 or RGF9 treatment. In contrast, RGI3 associates with BAK1 only in the presence of RGF9, but not flg22 (Stegmann et al. 2022), indicating a potential preference of RGI3 for BAK1 in the absence of FLS2. RGF9 perception promotes the flg22-induced FLS2-BAK1 complex formation and increases the FLS2 and EFR protein levels, likely through posttranslational modification (Stegmann et al. 2022). It is yet to be determined whether RGF9-induced immune complex formation and PRR protein accumulation are mechanistically linked.

**Fig. 2.**
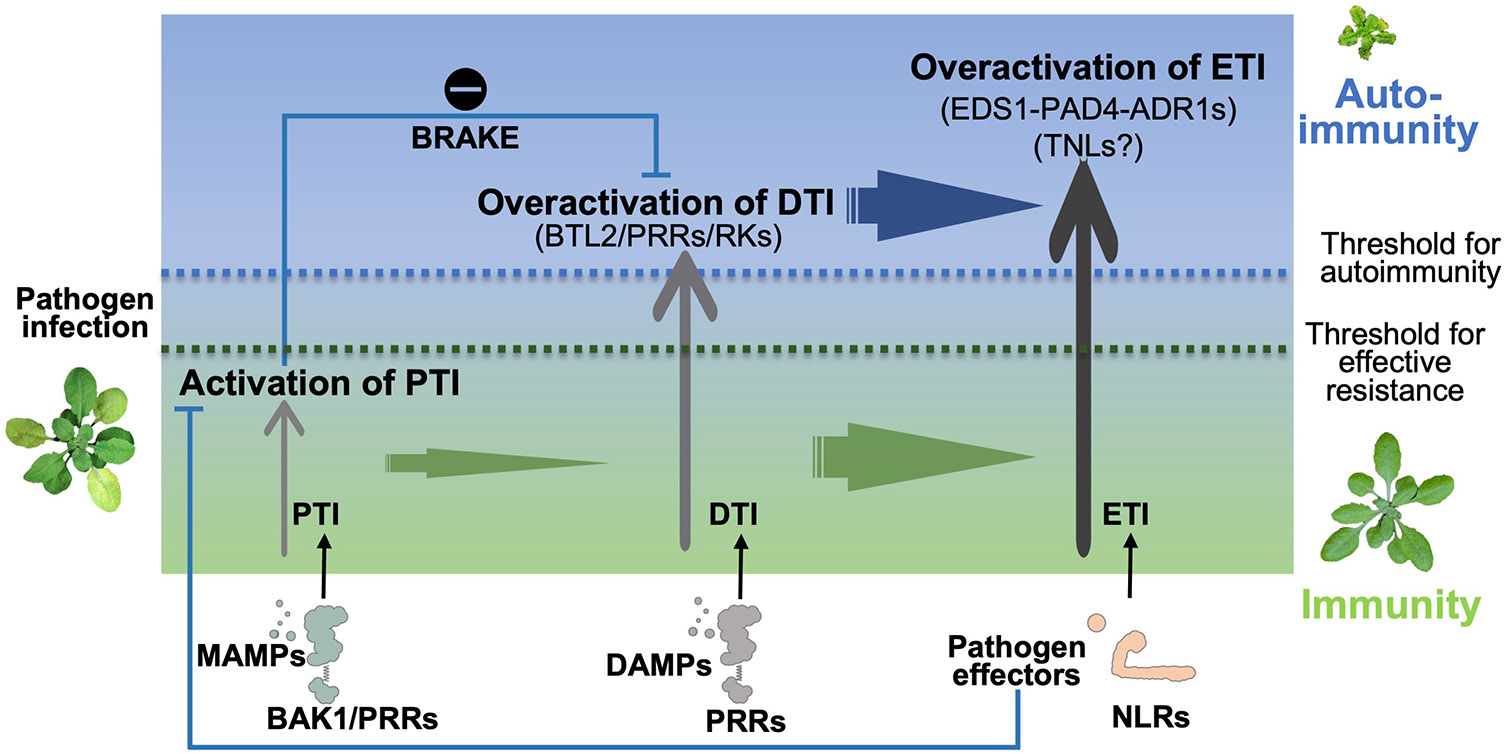
A model of BAK1/BTL2-mediated pattern-triggered immunity (PTI)–danger-triggered immunity (DTI)–effector-triggered immunity (ETI) interaction. When a pathogen invades, PTI serves as the primary defense mechanism against infections, and ETI is activated via the recognition of pathogen effectors by nucleotide-binding site leucine-rich repeat receptors (NLRs). BAK1 is an essential co-receptor of multiple pattern recognition receptors (PRRs) that initiate PTI. Pathogen-induced PTI stimulates the production of modified self-components known as danger-associated molecular patterns (DAMPs) and phytocytokines, which elicit DTI. DTI could amplify PTI and activate ETI to establish robust protection against pathogen infections. Overactivation of ETI could lead to autoimmunity. BAK1 is also essential to keep BTL2 inactive, which otherwise overactivates DTI and ETI. Thus, BAK1 has dual roles in activating PTI and restraining DTI and ETI. Some pathogens could secrete effectors that target BAK1 for degradation or perturbation, which leads to the disruption of PTI. Meanwhile, BTL2 is derepressed in the absence of BAK1, and associates with PRRs or other receptor kinases (RKs) to activate DTI. This activated DTI triggers EDS1-PAD4-ADR1-mediated ETI. It is likely that NLRs, especially TNLs (Toll/interleukin receptor domain-containing NLRs), are involved in the activation of the EDS1-PAD4-ADR1 module. Thus, BTL2-mediated DTI is a double-edged sword, which could compensate for the disrupted PTI when BAK1 is targeted by pathogens, but also could induce autoimmunity when BAK1 and its closest homolog, SERK4, are fully disrupted by pathogens. MAMPs, microbe-associated molecular patterns.

Because BAK1 and SERK4 are co-receptors for multiple PRRs, their integrity is central to maintaining immune homeostasis. Depletion of BAK1/SERK4 abolishes plant PTI, but triggers autoimmunity (He et al. 2007; Kemmerling et al. 2007). The LRR-RK BTL2 (BAK-TO-LIFE 2), identified as an autoimmunity suppressor of *bak1/serk4* from an interference RNA (RNAi)-based genetic screen, was shown to potentiate multiple phytocytokine-mediated DTI signaling, including SCOOPs and Pep1, which is otherwise suppressed by BAK1-mediated specific phosphorylation in wild-type plants (Yu et al. 2023). When the BAK1/SERK4 module is interrupted, BTL2 is markedly elevated, thereby unleashing the BAK1/SERK4 suppression on BTL2 for autoimmune elicitation (Yu et al. 2023). In line with this, BAK1 is targeted for degradation or perturbation by various pathogen effectors to dampen plant immunity (Li et al. 2016; Shan et al. 2008; Yamada et al. 2016; Zhou et al. 2014). Thus, in addition to their conventional role as shared co-receptors, BAK1/SERK4 constrain autoimmunity through a specific phosphor-switch on BTL2 to prevent overactivation of phytocytokine signaling (Fig. 2). BTL2 has likely evolved as a surveillance system and functions as an alternative co-receptor of multiple phytocytokine receptors in compensating immunity when PTI is compromised with the depletion or perturbation of BAK1/SERK4 co-receptors by pathogens. BTL2-conditioned compensatory immunity may also explain why Pep1-triggered immune response is relayed and potentiated in *bak1* mutants, although BAK1 is a co-receptor of Pep receptors PEPR1/PEPR2 (Yamada et al. 2016).

## RKs and RPs Contribute to the Unified PTI–DTI–ETI Immune Network

The plant innate immunity was traditionally divided into a two-layered system, comprising PRR-mediated PTI and NLR-mediated ETI (Jones and Dangl 2006). Upon pathogen infection, the first line of defense is initiated by the perception of MAMPs, which in turn triggers the production of DAMPs, leading to the activation of DTI. DTI plays a crucial role in reinforcing and amplifying PTI immune responses (Fig. 2). The signaling pathways mediated by PRRs and NLRs are interconnected through various convergent points with overlapping outputs. These include processes like ROS burst, MAPK activation, calcium influx, transcriptional reprogramming, and phytohormone signaling (Bernoux et al. 2022; Katagiri 2018; Ngou et al. 2022; Rhodes et al. 2022a; Thomma et al. 2011; Yuan et al. 2021b). This convergence blurs the distinction between PTI and ETI. Furthermore, RKs and RPs function as membrane-located surveillance sensors, regulating the PTI and DTI cascades in the context of ETI to ensure the effectiveness of the plant immune response as discussed in the sections “PTI potentiates ETI through DTI” and “The PTI-activated MAPK signaling suppresses NLR-triggered autoimmunity.”

### PTI and ETI mutually potentiate each other

Recent studies have provided compelling evidence demonstrating a mutual potentiation between PRR- and NLR-mediated immunity (Ngou et al. 2021; Pruitt et al. 2021; Tian et al. 2021; Yuan et al. 2021a). The activation of PRRs has been shown to enhance NLR-mediated HR (Ngou et al. 2021). For instance, the AvrRpt2-induced RPS2 activation, triggering HR development, was compromised in mutant plants lacking multiple PRRs (Yuan et al. 2021a). PRR signaling, beyond its role in HR development, is also essential for NLR-mediated MAPK activation and ROS production (Ngou et al. 2021; Yuan et al. 2021a). This conclusion is substantiated by the observation that disease resistance mediated by NLRs is compromised in plants with mutations in either PRRs or PRR co-receptors (Ngou et al. 2021; Yuan et al. 2021a). Consequently, the activation of PRRs is a requirement for NLR-mediated immunity. Reciprocally, the activation of NLRs amplifies and prolongs PRR-mediated immune responses (Ngou et al. 2021; Yuan et al. 2021a). Activation of NLRs leads to the accumulation of multiple PRR-signaling components such as BIK1, RBOHD (RESPIRATORY BURST OXIDASE HOMOLOG D), and MAPKs (Ngou et al. 2021; Yuan et al. 2021a). In summary, PTI and ETI mutually reinforce each other, facilitating robust and sustained defense responses against pathogen infections (Fig. 2). However, the precise underlying mechanisms require further exploration.

### PTI potentiates ETI through DTI

Plant PTI induces the production of DAMPs, which are recognized by their corresponding PRRs, culminating in the activation of DTI for a robust and balanced PTI. Recent studies propose that DTI may be a pivotal mechanism by which PTI amplifies ETI. Alongside the well-known MAMPs, DAMP molecules like Pep1 have also been recognized as contributors to ETI, triggering processes such as ROS production and autoimmunity (Ngou et al. 2021). This underscores the indispensable role of DAMPs in the ETI response. Furthermore, natural triggers of cell death, including HopB1 and pg23, induce BAK1 cleavage, which negatively influences PTI (Wu et al. 2020; Zhang et al. 2021). In such scenarios, the RK BTL2 emerges as a surveillance hub, effectively activating multiple phytocytokine signaling pathways in the absence of co-receptors BAK1/SERK4 (Yu et al. 2023). This activation results in autoimmunity, with a dependence on the EDS1 (ENHANCED DISEASE SUSCEPTIBILITY 1)-PAD4 (PHYTOALEXIN DEFICIENT4)-ADR1 (ACTIVATED DISEASE RESISTANCE 1) module (Yu et al. 2023). Additionally, CNGC (CYCLIC NUCLEOTIDE-GATED CHANNEL) calcium channels modulate Ca^2+^ homeostasis in the regulation of BAK1/SERK4-mediated autoimmunity (Yu et al. 2019). Notably, BTL2 phosphorylates and enhances the activity of the CNGC20 channel, further promoting cell death, thus emphasizing the importance of RKs in maintaining cellular Ca^2+^ homeostasis. Therefore, it is plausible that BTL2 functions as a molecular switch, working with phytocytokine-induced DTI to compensate for disrupted PTI, resulted from BAK1/SERK4 perturbation, thereby enhancing ETI defenses. Similarly, when BAK1 is inactivated, the LRR-RK BIR3 (BAK1-INTERACTING RECEPTOR-LIKE KINASE 3) directly interacts with the NLR protein CSA1 (CONSTITUTIVE SHADE AVOIDANCE 1), leading to the induction of CSA1-triggered ETI, which ultimately culminates in cell death (Schulze et al. 2022; Yang et al. 2022). Thus, it becomes evident that DTI signaling can be regarded as a crucial junction connecting plant PTI and ETI.

### The PTI-activated MAPK signaling suppresses NLR-triggered autoimmunity

MAPK cascades, composed of a three-tiered kinase system, play a pivotal role in both PTI and ETI activation (Meng and Zhang 2013; Rodriguez et al. 2010; Tena et al. 2011). Intriguingly, disruption of the MEKK1-MKK1/2-MPK4 cascade, activated by PRRs, leads to autoimmunity and cell death mediated by the NLR protein SUMM2 (suppressor of *mkk1 mkk2*) (Gao et al. 2008; Ichimura et al. 2006; Zhang et al. 2012). In this context, a VIGS (Virus-induced gene silencing)-based RNAi screen identified the cell surface-localized malectin-like RK LET1 (LE-TUM1) as a suppressor of cell death caused by silencing *MEKK1* (Liu et al. 2020). An additional malectin-like RK, LET2, functions synergistically with LET1 to regulate SUMM2-mediated autoimmunity (Huang et al. 2020; Y. Liu et al. 2021). LET2 complexes with LET1 and promotes LET1 phosphorylation (Huang et al. 2020).

MEKK2 and CRCK3 (CALMODULIN-BINDING RECEPTOR-LIKE CYTOPLASMIC KINASE 3) also play roles in regulating SUMM2 activation (Kong et al. 2012; Su et al. 2013; Zhang et al. 2017). LET1 and LET2, along with SUMM2, MEKK2, and CRCK3, form a multimeric complex (Huang et al. 2020; Liu et al. 2020; Y. Liu et al. 2021). MEKK2 acts as a scaffold and regulates SUMM2 proteostasis by affecting SUMM2 ubiquitylation and degradation (Liu et al. 2020). MEKK2 interacts with both LET1 and SUMM2, thereby stabilizing LET1 and SUMM2 for immune response activation (Liu et al. 2020). The precise mechanisms by which LET1 and LET2 activate SUMM2 remain unknown. However, considering that LET2 phosphorylates CRCK3 (Y. Liu et al. 2021), it is conceivable that a sequential phosphorylation module, consisting of the LET2-LET1-CRCK3 complex, may be monitored or sensed by SUMM2 to activate SUMM2-mediated immunity. In this case, PTI-activated MAPKs suppress the NLR SUMM2-triggered autoimmunity while serving as a guardee monitored by NLR. This seems to accord with the guard hypothesis, which was proposed to explain the potential mechanism of the indirect recognition between R and Avr after the gene-for-gene hypothesis (Dangl and Jones 2001) (Fig. 1).

## Concluding Remarks and Future Perspectives

More than 600 RKs and 57 RPs are predicted in the *Arabidopsis* genome, but the cognate ligands and physiological roles for most of them are unknown. Do all of them serve as sensors for pathogenic or danger threats? What are the common patterns or pathovar-specific ligands involved in such recognitions? RK/RP-initiated perceptions often go through unified signaling hubs, and additionally, RKs/RPs also participate in sensing environmental stresses and regulating growth and development. How do plants maintain signaling specificity in response to different stimuli under different contexts to ensure the execution of distinct biological processes? For instance, many channels are involved in calcium signaling upon elicitor perception. Is there a major channel for different types of elicitation, or do several channels contribute quantitatively to the process? Hypotheses to explain signal specificity could be related to the regulation in different tissues or cell types or at the cellular level, such as the existence of nanodomains in plasma membrane consisting of different composition of PRRs and regulators. Recent technical advancements like tissue-/site-specific gene editing, in situ spatial single-cell transcriptomics, and superresolution singlemolecule microscopy will facilitate deciphering the orchestrated interplay of RKs and RPs in sensing diverse stimuli.

DAMPs/phytocytokines are central regulators of immunity and growth, yet the processes underlying their maturation and transport remain elusive. Additionally, DTI and PTI share intersecting signaling pathways. Is DTI regulated in a manner similar to PTI concerning its activation and dampening mechanisms? The mechanistic link between PTI and ETI is being revealed, offering insight into the unified immune network, along with a new perspective on the role of DTI in this integrated system. In the case of BTL2, BTL2/DAMP-activated resistance is mediated by ETI nodes, which are also required for BTL2-induced cell death upon PTI disruption, suggesting a switch-on of DTI-ETI to compensate for the abolished PTI and a linking role of DTI between PTI and ETI. While DAMPs are transcriptionally upregulated upon BTL2 overexpression, the mechanisms through which BTL2 activates DTI and the involvement of ETI signaling are yet to be elucidated. Most recent advancements have been derived from studying the model plant *Arabidopsis*. However, their applicability to other plant species remains uncertain. Future research should leverage classic genetics and biochemistry, along with tools from bioinformatics, cell biology, and structural biology, to address the gaps in our understanding of the plant defense network.

Some RKs and RPs, along with their cognate phytocytokine ligands, are also involved in regulating plant growth and development. For instance, PEPs, PIPs, and SCOOPs inhibit primary root growth and lateral root formation (Rzemieniewski and Stegmann 2022). The exact nature of the growth suppression, either as an adaptive response or an active adjustment in the equilibrium between growth and responses to biotic and environmental factors, remains to be established. In addition, perception of phytocytokines by different receptors leads to distinct cellular and physiological responses in addition to immunity. RALF23, perceived by FER in modulating FLS2-mediated signaling and immunity (Stegmann et al. 2017), can be sensed by the RK ANJ (ANJEA)-FER complex inducing ROS production in the stigma papillae. The PCP-B (POLLEN COAT PROTEIN B)-class peptides compete with RALF23 and RALF33 for binding to the ANJ-FER complex, thereby reducing stigmatic ROS and allowing pollen hydration, a delicate signaling communication between pollen and stigma in controlling plant sexual reproduction (C. Liu et al. 2021). Moreover, stigma- and pollen-derived RALFs and their cognate RK *Cr*RLK1L receptor complexes have been implicated in pollen tube penetration for an intergeneric hybridization barrier (Lan et al. 2023). Therefore, RKs and RPs play a crucial role in coordinating responses to pathogenic invaders, as well as in regulating plant growth and development.

In addition to the regulation of expression and maturation of phytocytokines, layered mechanisms may contribute to RKs and RPs in eliciting largely overlapping signaling events while preserving signaling specificity. It has been reported that FLS2-BAK1 is affected by nanoscale mobility behaviors upon perception of RALF23 by FER (Gronnier et al. 2022). This suggests the role of nanodomains in the plasma membrane, which are composed of different proteins and lipids, in maintaining RK- and RP-mediated spatial and signaling specificity. Additionally, PEP1 perception inhibits primary root growth but promotes root hair formation and defense responses in leaves. Restricted expression of PEPR2, the PEP1 receptor in different root tissues, uncouples immune responses and root growth (Okada et al. 2021). These observations imply distinct signaling actions from different tissues, cell types, or even specific cells. A future challenge is to tease out the concerted operations among different cell types at the whole-organism level.

Flor’s study on flax and flax rust fungus, which displayed the Mendelian relationship governing resistance, established the gene-for-gene theory. Subsequent models, such as the guard hypothesis and the decoy model, provided explanations for indirect Avr-R protein interactions. Identification of general elicitors, without clear R-Avr specificity, led to the establishment of MAMP and PRR concepts. The zigzag model integrated MAMPs and Avrs, distinguishing two layers of recognition and immunity. Additionally, DAMPs were introduced as endogenous immune response inducers. More recently, PTI–ETI and PTI–DTI–ETI are considered an integrated system (Fig. 1). Overall, the genetic and molecular dissection of multilayered defense systems encompassing PTI, DTI, and ETI and their regulation has greatly broadened Flor’s gene-for-gene concept.
